# Supplementary material for: The genome sequence of Schoenoplectus triqueter (L.) Palla, 1888 (Poales: Cyperaceae)
Source: Wellcome Open Res. 2026 Jun 25;11:382. [Version 1] doi: 10.12688/wellcomeopenres.26828.1 (PMC13555089; doi:10.12688/wellcomeopenres.26828.1)
Supplement: Peer review history

# Reviewer report: Reviewer response for version 1

**DOI:** 10.21956/wellcomeopenres.29542.r160383

**Published:** 2026-08-03

**Authors:** Nguyen Nhat Nam

**Affiliations:** 1 Tra Vinh University, Vinh Long, Vietnam

This study reports a reference genome for Schoenoplectus triqueter (Triangular Club-rush; Streptophyta, Magnoliopsida, Poales, Cyperaceae). The assembled nuclear genome spans 580.26 Mb, with 98.75% of the sequence assigned to 21 chromosome-scale pseudomolecules. The plastid genome and five mitochondrial sequences were also recovered. Ensembl annotation identified 31,746 protein-coding genes. Overall, the manuscript is clearly written, well-defined methodology approach and high-quality genomic resource results. Therefore, it can be accepted for consideration for indexing.

Are sufficient details of methods and materials provided to allow replication by others?

Yes

Is the rationale for creating the dataset(s) clearly described?

Yes

Are the datasets clearly presented in a useable and accessible format?

Yes

Are the protocols appropriate and is the work technically sound?

Yes

Reviewer Expertise:

Plant molecular biology and genomics

I confirm that I have read this submission and believe that I have an appropriate level of expertise to confirm that it is of an acceptable scientific standard.

# Reviewer report: Reviewer response for version 1

**DOI:** 10.21956/wellcomeopenres.29542.r160382

**Published:** 2026-07-22

**Authors:** Sheng-Kai Hsu

**Affiliations:** 1 Cornell University, Ithaca, NY, USA

This Data Note reports a chromosome-level genome assembly for *Schoenoplectus triqeter *along with its annotation. The sequencing and assembly workflow (PacBio HiFi with hifiasm, purge_dups, Hi-C scaffolding in YaHS, ASCC decontamination, and manual curation) is the established and appropriate ToL approach. Base-level quality is excellent (combined QV 63.5; primary QV 64.3), contiguity is strong (contig N50 3.48 Mb, scaffold N50 27.80 Mb), and 98.75% of the assembly is assigned to 21 chromosomal pseudomolecules supported by a clean Hi-C contact map.

 General comments:

 1. Standard BUSCO poales_odb10 reference set seems to be consistently problematic for Cyperaceae genomes (see also another recent genome report on *Carex sylvatica*: https://wellcomeopenresearch.org/articles/11-332). This may be worth of some discussion and revisit the poales BUSCO reference list.

 2. The assembly was annotated by Ensembl with standardized pipeline. For a general audience, it would be recommended to provide a brief description on the annotation pipeline. One would need to know whether the RNASeq data from leaves is the only input to generate the annotation and decide if he/she would like to reannotate it with data from more tissues or adapt an ab initio RNASeq-free pipeline.

Are sufficient details of methods and materials provided to allow replication by others?

Yes

Is the rationale for creating the dataset(s) clearly described?

Yes

Are the datasets clearly presented in a useable and accessible format?

Yes

Are the protocols appropriate and is the work technically sound?

Yes

Reviewer Expertise:

phylogenomics

I confirm that I have read this submission and believe that I have an appropriate level of expertise to confirm that it is of an acceptable scientific standard.

# Reviewer report: Reviewer response for version 1

**DOI:** 10.21956/wellcomeopenres.29542.r159492

**Published:** 2026-07-14

**Authors:** María Sanz-Arnal

**Affiliations:** 1 Biology, Rey Juan Carlos University, Móstoles, Community of Madrid, Spain

The authors present the annotated genome from a cultivated specimen of S *choenoplectus triqueter* (Cyperaceae). The data were generated using PacBio HiFi long-read sequencing, Hi-C scaffolding, and RNA sequencing, following the standard procedures for eukaryotic genome assemblies established at the Darwin Tree of Life Consortium.

 The assembly demonstrates strong technical quality, with 98.75% of the genome anchored to 21 chromosomal pseudomolecules, high consensus quality (QV 63.5), and comprehensive gene annotation comprising 31,746 protein-coding genes.

 Please find some comments and corrections.  

**General comments:**

 (1) Could the authors comment on the relatively low BUSCO completeness (75.6%, with 32.8% duplicated) compared to typical DToL assemblies of similar quality? Could this reflect the suitability of the *poales_odb10* reference set for Cyperaceae?

 (2) The text reports chromosome counts of 2n = 40 and 42, while the 21 recovered pseudomolecules would imply n = 21 (consistent with 2n = 42). Does this discrepancy reflect cytotype variation within the species, or an error in the earlier cytological literature?

 (3) In Methods, revise how equipment and protocols are cited. Several pieces of equipment mentioned throughout the Methods (e.g., Megaruptor 3, FemtoPulse system, Nanodrop spectrophotometer) are cited without full manufacturer details (company, city, country). Could the authors add this information?

**Minor comments:**

 (1) Consider moving the following paragraph to the end of the Background section: "Here we present a chromosomally complete genome (...) collected at Higher Ferry Farm, Devon, United Kingdom.";

 (2) Comma:  "In Ireland *S. triqueter* is still present..." should read "In Ireland **,** *S. triqueter* is still present...";

 (3) Typo: "MSB nursey.)" should read "MSB nursery)."; and

 (4) consider rephrasing this sentence to avoid redundancy: "For HMW DNA extraction, 53.5 mg of leaf tissue was used. Tissue from the leaf was homogenised by..." could be e.g., "For HMW DNA extraction, 53.5 mg of leaf tissue was homogenised by...".

**Overall assessment**: The methods are clearly presented, and the quality of the data is well demonstrated. No major issues are to report; the points above are intended to improve clarity.

Are sufficient details of methods and materials provided to allow replication by others?

Yes

Is the rationale for creating the dataset(s) clearly described?

Yes

Are the datasets clearly presented in a useable and accessible format?

Yes

Are the protocols appropriate and is the work technically sound?

Yes

Reviewer Expertise:

Cyperaceae, Plant systematic and phylogenomic

I confirm that I have read this submission and believe that I have an appropriate level of expertise to confirm that it is of an acceptable scientific standard.
